# Mental Disorders and Suicidality in Transgender and Gender-Diverse People

**DOI:** 10.1001/jamanetworkopen.2024.36883

**Published:** 2024-10-02

**Authors:** Heidi Eccles, Alex Abramovich, Karen A. Patte, Tara Elton-Marshall, Nicole Racine, Mark A. Ferro, Jordan Edwards, Kelly K. Anderson, Tracie O. Afifi, Marie-Claude Geoffroy, Mila Kingsbury, Ian Colman

**Affiliations:** 1 School of Epidemiology and Public Health, University of Ottawa, Ottawa, Ontario, Canada; 2 Institute for Mental Health Policy Research, Centre for Addiction and Mental Health, Toronto, Ontario, Canada; 3 Department of Health Sciences, Brock University, St Catharines, Ontario, Canada; 4 Department of Psychology, University of Ottawa, Ottawa, Ontario, Canada; 5 School of Public Health Sciences, University of Waterloo, Waterloo, Ontario, Canada; 6 Department of Psychiatry and Behavioural Neurosciences, McMaster University, Hamilton, Ontario, Canada; 7 Department of Epidemiology and Biostatistics, Schulich School of Medicine and Dentistry, Western University, London, Ontario, Canada; 8 Departments of Community Health Sciences and Psychiatry, Max Rady College of Medicine, University of Manitoba, Winnipeg, Manitoba, Canada; 9 Department of Psychiatry, Douglas Mental Health University Institute, McGill Group for Suicide Studies, McGill University, Montreal, Quebec, Canada

## Abstract

This cross-sectional study examines the prevalence and risk of mental disorders, substance use disorders, and suicidal behaviors among transgender and gender-diverse (TGD) and cisgender Canadians.

## Introduction

Marginalized populations, such as people who identify as transgender and gender diverse (TGD), carry a disproportionately high burden of mental disorders, compared with the general population.^1,2^ It is well documented that TGD people are more likely to face trauma and adversity including physical attacks, verbal abuse, stigma, and discrimination^3^ compared with cisgender people. Although there are studies reporting on mental disorders in the TGD population, there are few large population-based studies that compare the TGD population with the cisgender population using a validated diagnostic tool.^4^ The objectives of this study were to investigate the prevalence and risk of mental disorders, substance use disorders, and suicidal behaviors (both past 12 months and lifetime) in a population-representative sample of TGD and cisgender Canadians.

## Methods

This study uses data from the Mental Health and Access to Care Survey (MHACS), which is a nationally representative cross-sectional study administered by Statistics Canada from March to July 2022. Statistics Canada obtained informed consent from all participants; ethical approval for this specific analysis was not required. The STROBE reporting guideline was followed.

There was a 25% response rate, resulting in a sample size of 9861 people. When sex at birth and gender identity matched, respondents were classified as cisgender; in the case of a mismatch, respondents were classified as TGD. Past 12-month and lifetime major depressive episode, generalized anxiety disorder, bipolar disorder, social phobia, alcohol use disorder, and substance use disorder, suicidal thoughts, plan, and attempt were assessed as outcomes (eAppendix in Supplement 1). To ensure that the sample was representative of the Canadian population, analyses were weighted using survey and bootstrap weights provided by Statistics Canada. Modified Poisson regression with sandwich error variance estimation was used to estimate the association between gender identity and mental health outcomes. Age, household income, chronic physical illness, and racial or ethnic minority status were included in the regression model to adjust for confounding. All analysis was completed in Stata version 18 (StataCorp). Statistical significance was assessed by *P* < .05 and 95% CIs that did not include 1.

## Results

Of the total sample of 9861, 52 (0.53%) identified as TGD; demographic data can be found in Table 1. Past 12-month and lifetime major depressive episode, generalized anxiety disorder, bipolar disorder, social phobia, substance use disorder, suicide ideation, suicide plan, and suicide attempts were higher in TGD respondents compared with cisgender respondents (eg, lifetime prevalence of depression among cisgender: 13.7% [95% CI, 12.9%-14.6%] vs TGD: 63.7% [95% CI, 46.6%-78.0%]; adjusted rate ratio, 2.78 [95% CI, 2.16-3.57]) (Table 2). After adjusting for confounders, the risk of all 12-month and past-year mental disorders, substance use disorder, and suicide ideation were higher in TGD respondents compared with cisgender participants. The risk of lifetime suicide plan and attempts was significantly higher in TGD respondents. Alcohol use disorder did not differ between the groups (past 12 months or lifetime).

**Table 1.** Demographic Information About the Study Sample

Variable	Full sample	Cisgender	Transgender and gender diverse
Age, mean (SE), y	47.6 (0.2)	47.7 (0.2)	29.3 (1.8)
Income, mean (SE), $	125 604.0 (1300.9)	125 706.7 (1306.1)	109 168.1 (13 363.6)
Racial or ethnic minority, % (95% CI)^a^			
Yes	28.3 (27.3-29.3)	28.3 (27.3-29.3)	24.4 (13.4-40.3)
No	71.7 (70.7-72.7)	71.7 (70.7-72.7)	75.6 (59.7-86.6)
Chronic disease, % (95% CI)	54.4 (53.2-55.6)	54.4 (53.2-55.6)	53.7 (36.6-69.9)

^a^ Racial or ethnic minority groups included Arab, Black, Chinese, Filipino, Japanese, Korean, Latin American, South Asian, Southeast Asian, West Asian, and other.

**Table 2.** Weighted Prevalence of Psychiatric Disorders in the Past 12 Months and Lifetime and Poisson Regression Analysis for Mental Health Outcomes Associated With Gender Status

Outcome	Past 12 mo			Lifetime		
	Prevalence cisgender (95% CI)	Prevalence TGD (95%CI)	Adjusted RR (95% CI)^a^	Prevalence cisgender (95% CI)	Prevalence TGD (95% CI)	Adjusted RR (95% CI)^a^
Depression	7.3 (6.7-8.1)	40.9 (24.4-59.7)	3.04 (2.01-4.59)	13.7 (12.9-14.6)	63.7 (46.6-78.0)	2.78 (2.16-3.57)
Bipolar disorder	2.1 (1.7-2.5)	13.5 (5.2-30.9)	3.22 (1.25-8.31)	3.3 (2.9-3.8)	18.3 (7.8-37.3)	2.91 (1.30-6.55)
Anxiety	5.1 (4.5-5.7)	29.1 (14.8-49.1)	3.01 (1.68-5.40)	13.1 (12.3-14.0)	52.5 (34.7-69.7)	2.54 (1.88-3.41)
Social phobia	6.9 (6.3-7.6)	37.5 (21.9-56.2)	2.77 (1.70-4.50)	14.3 (13.4-15.2)	72.5 (55.2-85.0)	2.95 (2.38-3.66)
Suicide ideation	3.5 (3.1-4.0)	25.2 (12.3-44.9)	3.48 (1.78-6.78)	11.1 (10.3-11.9)	62.2 (44.6-77.1)	3.40 (2.50-4.63)
Suicide plan	1.0 (0.7-1.3)	NA^b^	1.11 (0.24-5.22)	4.8 (4.3-5.3)	27.8 (15.0-45.6)	3.39 (1.85-6.19)
Suicide attempt	0.4 (0.2-0.6)	NA^b^	1.72 (0.22-13.24)	3.0 (2.6-3.5)	32.6 (17.8-52.0)	6.22 (3.72-10.41)
Alcohol use disorder	2.2 (1.8-2.6)	NA^b^	0.83 (0.12-5.56)	16.6 (15.6-17.6)	22.8 (10.3-43.1)	1.21 (0.61-2.38)
Substance use disorder	1.8 (1.5-2.2)	16.0 (5.5-38.3)	4.19 (1.71-10.27)	8.4 (7.7-9.2)	26.5 (13.0-46.7)	1.86 (1.01-3.46)

Abbreviations: RR, risk ratio; TGD, transgender and gender diverse.

^a^ Adjusted for income, age and ethnic minority status, chronic physical illness.

^b^ Prevalence not reported due to Statistics Canada rules regarding release of small cell counts.

## Discussion

To our knowledge, this is one of the first population-based studies to find higher prevalence of mental disorders and suicidal behavior in TGD people compared with the cisgender population. This finding aligns with other studies, which have found significantly higher rates of mental health–related health service use among transgender people compared with the general population.^1,5^ This disparity may be explained by minority stress theory, which posits that the experience of prejudice and negative social experiences by members of historically stigmatized groups can have substantial impacts on both physical and mental health.^6^ Further research should investigate what factors lead to this and what interventions may mitigate this inequity. Limitations of this study were the small sample of TGD people and the cross-sectional nature of the study. It is also unclear whether TGD people were less likely to participate in the study compared with others. There could also be residual confounding because we were unable to include additional covariates due to sample size.

## Notes

Supplement 1.
eAppendix.

Supplement 2.
Data Sharing Statement
