# Supplementary material for: Mental Disorders and Suicidality in Transgender and Gender-Diverse People
Source: JAMA Netw Open. 2024 Oct 2;7(10):e2436883. doi: 10.1001/jamanetworkopen.2024.36883 (PMC11447565; doi:10.1001/jamanetworkopen.2024.36883)
Supplement: Supplement 1. — eAppendix. [file jamanetwopen-e2436883-s001.pdf]

Supplemental Online Content

Eccles H, Abramovich A, Patte KA, et al. Mental disorders and suicidality in transgender and gender-diverse people. *JAMA Netw Open.* 2024;7(10):e2436883.
doi:10.1001/jamanetworkopen.2024.36883

eAppendix

This supplemental material has been provided by the authors to give readers additional information about their work.

© 2024 Eccles H et al. *JAMA Network Open*.

eAppendix

Methods:

This study uses the data from the Mental Health and Access to Care Survey (MHACS) collected in March-July 2022. MHACS is a nationally representative cross-sectional study of mental health by Statistics Canada. MHACS has a target sample of people living in Canada, aged 15 to 85 years residing in the 10 provinces. People living in institutions, on First Nations settlements and full-time members of the Canadian forces were excluded (approximately 3% of the population). Completion of the electronic questionnaire was assisted by a telephone interviewer.

Exposure: The exposure of interest was TGD status. This was defined using two questions: 1) “What was your sex at birth?” (response options “Male” and “Female) and 2) “What is your gender?” (participants could select “Male”, “Female,” or specify another gender). When sex at birth and gender matched, respondents were classified as cisgender; in the case of a mismatch respondents were classified as TGD. This 2-step question has high agreement with tools that include multi-dimensional questions and is easily understood by both TGD and cisgender people. If a person did not answer the gender question (I don’t know or refusal) but did answer the sex at birth question, then they were classified as cisgender.

Outcomes: A modified version of the computer-administered World Health Organization Composite International Diagnostic Interview was used to assess past 12-month and lifetime major depressive episode, generalized anxiety disorder, bipolar disorder, social phobia, alcohol use disorder, and substance use disorder. Suicidal thoughts were assessed with the question, “In the past 12 months, did you seriously think about suicide or taking your own life?”; suicidal plans with “Did you make a plan for attempting suicide at any time in the past 12 months?”; and, suicide attempts with “During the last 12 months, did you attempt suicide or try to take your own life?”. Lifetime suicide ideation, plan, and attempts were additionally examined with similar questions.

© 2024 Eccles H et al. *JAMA Network Open*.
