# Supplementary material for: Mental Disorders and Suicidality in Transgender and Gender-Diverse People
Source: JAMA Netw Open. 2024 Oct 2;7(10):e2436883. doi: 10.1001/jamanetworkopen.2024.36883 (PMC11447565; doi:10.1001/jamanetworkopen.2024.36883)
Supplement: Supplement 2. — Data Sharing Statement [file jamanetwopen-e2436883-s002.pdf]

Data Sharing Statement

Eccles. Mental Disorders and Suicidality in Transgender and Gender-Diverse People. *JAMA Netw Open*. Published October 02, 2024. doi:10.1001/jamanetworkopen.2024.36883

Data
Data available: No

Additional Information
Explanation for why data not available: Data for this study are made freely available for researchers through Statistics Canada's Research Data Centre program
